# Lactate modulates cardiac gene expression in mice during acute physical exercise

**DOI:** 10.1590/1414-431X2022e11820

**Published:** 2022-04-27

**Authors:** T.F. Cunha, J.S. Vieira, J.B. Santos, M.A. Coelho, P.C. Brum, D. Gabriel-Costa

**Affiliations:** 1 Escola de Educação Física e Esporte, Universidade de São Paulo, São Paulo, SP, Brasil; 2 Universidade Paulista, São Paulo, SP, Brasil; 3 Universidade da Força Aérea, Força Aérea Brasileira, Rio de Janeiro, RJ, Brasil

**Keywords:** Lactate, Cardiac gene expression, Mice, Physical exercise, Metabolism

## Abstract

The aim of the present study was to verify the role of lactate as a signaling molecule in cardiac tissue under physiological conditions. C57BL6/J male mice were submitted to acute running bouts on a treadmill at different exercise intensities (30, 60, and 90% of maximal speed - Smax) under the effect of two doses (0.5 and 5 mM) of α-cyano-4-hydroxycynnamate (CINN), a blocker of lactate transporters. Cardiac lactate levels, activity of the enzymes of glycolytic [hexokinase (HK) and lactate dehydrogenase (LDH)] and oxidative metabolism [citrate synthase (CS)], and expression of genes also related to metabolism [*LDH*, nuclear factor erythroid 2-related factor 2 (*NRF-2*), cytochrome oxidase IV (*COX-IV*), and peroxisome proliferator-activated receptor gamma coactivator 1-alpha (*PGC-1α*)] were evaluated. Elevated cardiac lactate levels were observed after high intensity running at 90% of Smax, which were parallel to increased activity of the HK and CS enzymes and mRNA levels of *PGC-1α* and *COX-IV.* No changes were observed in cardiac lactate levels in mice running at lower exercise intensities. Interestingly, prior intraperitoneal administration (15 min) of CINN (0.5 mM) significantly reduced cardiac lactate concentration, activities of HK and CS, and mRNA levels of *PGC-1α* and *COX-IV* in mice that ran at 90% of Smax. In addition, cardiac lactate levels were significantly correlated to both *PGC-1α* and *COX-IV* cardiac gene expression. The present study provides evidence that cardiac lactate levels are associated to gene transcription during an acute bout of high intensity running exercise.

## Introduction

Lactate is recognized as a metabolic integrating substrate, which supplies energy to the tissues and organs during physical exercise enabling the maintenance of performance (1-3). This energy integration is only feasible because lactate is able to cross cellular membranes to act in autocrine, paracrine, and endocrine manners in different cells and tissues. The transport of lactate across plasma membranes is carried out by a protein transport system, which consists of different isoforms of monocarboxylate transporters (MCT) (4). They are symport carriers of monocarboxylates (i.e., lactate, pyruvate, acetate) and are associated with proton influx (5,6). Due to the dual transport characteristic of MCT, lactate is able to be shuttled across the membranes in both directions, into or out of the cells, reaching the bloodstream and the organs. Especially during physical exercise, skeletal muscle cells are the main source of lactate production, which is driven by the bloodstream to be used as an energy substrate in other tissues, such as the heart (2). Thus, physical exercise can be an efficient strategy to increase the concentration of cardiac lactate, which is dependent on the intensity of the exercise. Indeed, cardiac lactate levels increase during high intensity physical exercise (7).

Cardiac cells use lactate as an energy substrate during exercise (2). When blood lactate concentration increases, lactate is shuttled to cytoplasm and mitochondrial matrix through MCT1, which is the main MCT isoform expressed in cardiac membranes (sarcoplasmic and inner-mitochondrial) (6,8). Once into mitochondrial matrix, it is oxidized with co-participation of the mitochondrial isoform of lactate dehydrogenase (mLDH) (2). The use of lactate as a metabolic substrate enables the heart to save its glycogen stocks, increasing energy capacity (6,9). Although most of the lactate produced during exercise is oxidized and used as energy substrate by for the heart, lung, and brain (2), some is converted to glucose by the liver and kidneys (∼25%). Lactate-induced gluconeogenesis controls blood glucose levels and helps maintain performance, especially during high-intensity prolonged exercise and in the fasting condition (10).

Additionally, lactate is also an important signaling molecule in metabolism. Indeed, lactate increases gene expression and protein synthesis of lactate oxidation complex (LOC: molecules involved in lactate and oxidative metabolism), such as the proliferator receptor coactivator type 1 alpha (*PGC-1α*), nuclear factor erythroid-2 related factor (*NRF-2*), and cytochrome oxidase IV (*COX-IV*) in skeletal muscle cell lineages (11). A similar effect was observed in isolated rat hearts with an increased expression of LOC genes (12). However, to date, the role of lactate in the modulation of genes and proteins in the heart has not been investigated *in vivo*.

Therefore, the aim of the present study was to evaluate the effect of lactate on the expression of genes related to energy and lactate metabolism in the heart.

## Material and Methods

### Ethical approval

This study was conducted according to ethical principles in animal research adopted by the Brazilian Council for the Control of Animal Experimentation (CONCEA) and approved by the Ethics and Research Committee of the University of São Paulo, Brazil (#2011/55).

### Study sample

Five-month-old male C57BL/6J mice (n=48) were maintained at the Laboratory of Cellular and Molecular Exercise Physiology (University of São Paulo) and housed three to five per cage in a temperature-controlled room (22°C) with a 12-h light/dark cycle. The animals had free access to standard chow and tap water.

### Graded treadmill exercise test and acute running session

Maximal running speed (Smax) was obtained according to the protocol of Ferreira et al. (13). Five days before testing, the mice were adapted to the treadmill by walking and running at low speeds for 10 min per day. The graded treadmill exercise test started at 6 m/min and exercise intensity was increased by 3 m/min (6-39 m/min) every 3 min at 0% grade until exhaustion (i.e., the mice were no longer able to run).

The Smax achieved in the maximal test was used to calculate the relative intensities for the acute running sessions with a constant workload of 30, 60, and 90% of Smax.

### Experimental design

**Figure 1:**
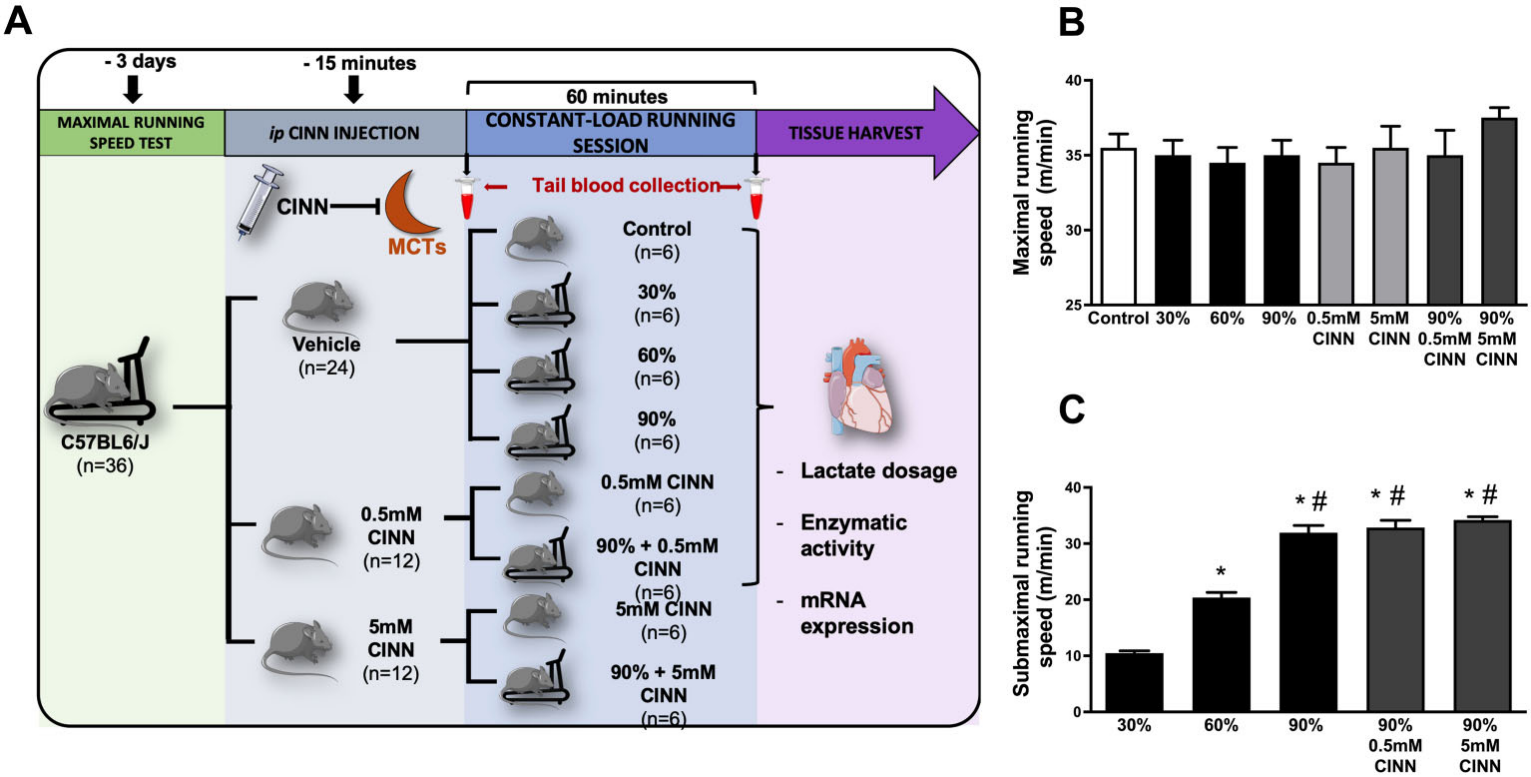
Experimental design (**A**), treadmill running speeds attained at the last stage of the graded treadmill exercise test (**B**), and the submaximal acute exercise session (**C**) of the groups running at 30%, 60%, and 90% of maximal speed, and 90% of maximal speed plus CINN (0.5 and 5 mM). Control, 0.5 mM CINN, and 5 mM CINN groups did not perform the submaximal acute exercise session. Data are reported as means±SE. *P<0.05 *vs* 30%, ^#^P<0.05 *vs* 60% (ANOVA). CINN: α-cyano-4-hydroxycynnamate.

The experimental design of the study is shown in Figure 1A. After the graded treadmill exercise test, mice were randomly assigned to four experimental groups (n=6/group) as follows: control, and animals that ran at 30%, 60%, and 90% of Smax. The control group was maintained at the treadmill, but it did not perform any exercise. The 30, 60, and 90% groups ran at constant intensity until exhaustion or until completing 60 min.

The same procedures were carried out with two other groups (n=6/group) that performed an acute running session at 90% of Smax. They received 0.5 or 5 mM of α-cyano-4-hydroxycynnamate (CINN), a blocker of lactate transporters (Sigma Aldrich, USA), administered intraperitoneally (*ip*) 15 min before the beginning of the running session. The respective controls that received both doses of CINN and did not perform physical exercise were also evaluated (n=6/group). These groups were assigned as: 90% 0.5 mM CINN group (treated with 0.5 mM of CINN and that ran at 90% of Smax); 90% 5 mM CINN group (treated with 5 mM of CINN and that ran at 90% of Smax); 0.5 mM CINN (treated with 0.5 mM of CINN and that did not perform any physical exercise); and 5 mM CINN (treated with 5 mM of CINN and that did not carry out any physical exercise). All control groups received the same volume of distilled water as the treated group, which corresponded to the highest dose of CINN (5 mM).

CINN was used in the present study in an attempt to develop a model in which the administration of the blocker would diminish lactate transport to the heart, preventing the increase of intracellular lactate levels. This increase of lactate levels in the heart is generally observed during physical exercise, allowing the comparison between groups that received the blocker and the groups that did not receive the blocker, but performed physical exercise. Since there is no other *in vivo* study that tested the effect of CINN on cardiac lactate transport (neither when administered via *ip* nor during physical exercise), we decided to set two concentrations that were used in previous *in vitro* studies and directly observe the response of lactate levels in cardiac tissue. The lowest concentration of CINN (0.5 mM) was applied because it corresponded to the IC_50_ for lactate transport observed in MCT1 expressed in Xenopus oocytes (14) and in isolated rat cells (15). On the other hand, the highest concentration of CINN (5 mM) was used because it elicited maximal inhibition of lactate uptake in cardiomyocytes of rats (15) and guinea pigs (16).

A stock solution of 30 mM of CINN was dissolved in distilled water and different volumes of this solution were administered (20-300 µL) to mice in order to attain the desirable concentrations of CINN (0.5 and 5 mM).

### Lactate concentration in the blood

Blood from the tail vein of the mice was collected at rest and at the end of the acute running session. The samples (25 µL) were treated with 1% (w/v) of sodium fluoride (Sigma Aldrich), and the lactate concentration was analyzed using an electroenzymatic method with the lactate analyzer YSI 2300 Stat Analyzer (Yellow Springs Instruments, USA).

### Lactate concentration in cardiac tissue

Immediately after the end of the acute running session, the mice were anesthetized and euthanized, and the left ventricle was dissected in a container surrounded with ice. The left ventricle tissues were transferred to 1.5-mL tubes and stored at -90°C.

The determination of cardiac lactate concentration was based on the Rosenberg and Rush technique and conducted as previously described by Gabriel-Costa et al. (12). Briefly, the left ventricles were homogenized with perchloric acid (3%, v/v) and centrifuged for 20 min at 4°C at 10,000 *g* to decrease protein content of the samples. The supernatant was used to measure lactate concentration. To determine the lactate level, the samples were first incubated with 0.2 M of glycine-semicarbazide buffer and 0.02 M of NAD^+^ (Sigma Aldrich) and the absorbance was measured in a wavelength of 340 nm (R1). Then, the preparations were incubated for 60 min at 40°C with LDH (2 mg/mL) obtained from bovine heart (Sigma Aldrich). Thereafter, a second reading was obtained at the same wavelength (R2). The values were used to calculate the net absorbance, with the following formula A = [R2 - (0.9.R1)] - [(B2 - (0.9.B1)], where A is the net absorbance, B1 is the blank absorbance without LDH, and B2 is the blank absorbance with LDH. The concentration of lactate in mM was derived from the values of net absorbance in a standard curve obtained between absorbance and increasing concentrations of lactate.

### RNA extraction and quantitative real time RT-PCR

Total RNA was isolated from left ventricle samples using Trizol (Invitrogen, USA). The RNA concentration and purity (260:280 nm ratio) was assessed in a spectrophotometer (Nanodrop 2000, Thermo Scientific, USA) and integrity was observed in agarose gel (2%, w/v) electrophoresis. The cDNA was synthesized using 1 μg of total RNA in a reaction including oligo dT (500 μg/mL), 10 mM of each dNTP, 5× first-strand buffer, 0.1 M DTT, ribonuclease inhibitor, and 200 μM of reverse transcriptase Superscript II (Invitrogen). The genes analyzed were: *LDH*, *NRF-2*, *COX-IV*, *PGC1α*, and cyclophilin, which was used as a reference gene. All primers were synthesized by Fermentas (USA) and their sequences are shown in Table 1. The amplifications were performed separately using SYBR Green/ROX qPCR Master Mix (Fermentas) in ABI Prism 5700 Sequence Detection System (Applied Biosystems Inc., USA).

**Table 1:** qRT-PCR primer sequences.

Gene	Forward	Reverse
*LDH*	5′-GCAGCAGGGTTTCTATGGAG-3′	5′-TGGAGACAGTGGGATTGTCA-3′
*NRF-2*	5′-GCACTCTGTGGAGTCTTCCATTTA-3′	5′-GAAGAATGTGTTGGCTGTGCTTTA-3′
*COX-IV*	5′-GAACAAGGGCACCAATGAGT-3′	5′-GTTGACCTTCATGTCCAGCA-3′
*PGC-1α*	5′-AAACTGCAGATTTGATGGACC-3′	5′-TTTCCCTCTTCAGCATAGTTC-3′
Cyclophilin	5′-TGGCAAGCATGTGGTCTTTGGGAAG-3′	5′-GGTGATCTTCTTGCTGGTCTTGCCATTC-3′

*LDH*: lactate dehydrogenase; *NRF2*: nuclear factor erythroid-2 related factor 2; *COX-IV*: cytochrome oxidase IV; *PGC-1α*: peroxisome proliferator-activated receptor gamma coativator-1 alpha.

Results were obtained using the comparative cycle threshold (Ct) method as described by the manufacturer. The ΔCt obtained from the subtraction of target gene and the reference gene (cyclophilin) Ct's was used to calculate de ΔΔCt from treatment groups in relation to the control group. The expression values were calculated with 2^-ΔΔCt^. Control group levels were arbitrarily set to 1.

### Enzyme activities

Assays for enzyme activities were conducted as previously described by Gabriel-Costa et al. (12). Left ventricle homogenates were obtained by mincing thawed tissues with specific ice-cooled buffers and centrifuged at 10,000 *g* for 20 min at 4°C.

#### Hexokinase

Left ventricle homogenates were obtained mincing the tissue in Tris-HCl buffer containing (in mM): 75 Tris-HCl, 7.5 MgCl_2_, 0.8 EDTA, 1.5 KCl, and 4 mercapto-ethanol, pH 7.5. After centrifugation, the supernatant was used to measure hexokinase (HK) activity. HK activity was obtained by monitoring the reduction of NADPH^+^ via coupled reactions. The working solution used in this assay was (in mM): 75 Tris-HCl, 7.5 MgCl_2_, 0.8 EDTA, 1.5 KCl, 4 mercapto-ethanol, 0.4 NADP+, 2.5 ATP, 1.4 units/mL of creatine phosphate, 0.05% (v:v) Triton X-100, excess G6PDH, and the sample. The reaction was started with 1 mM of glucose and monitored for 20 min at 25°C and 340 nm.

#### Lactate dehydrogenase

Left ventricle homogenates were obtained as indicated for the HK protocol. After centrifugation, the supernatant was used to measure LDH activity. The assay was based on monitoring oxidation NADH in the presence of pyruvate. The working solution used in the protocol was (in mM): 75 Tris-HCl, 1000 EDTA, and 2 NADH+H^+^. Sodium pyruvate (10 mM) was added after an incubation period of 10 min at 37°C and the reaction was monitored for 5 min at 340 nm.

#### Citrate synthase

Left ventricle homogenates were obtained by mincing the tissue in phosphate buffer containing (in mM): 50 sodium chloride and 1 EDTA, pH 7.4. After centrifugation, the supernatant was used to measure CS activity. The assay was based on the reaction of CoASH with dithiobis nitrobenzoic (DTNB) dye. The working solution used in the protocol was (in mM): 100 Tris-base, 1EDTA, 0.2 DTNB, 0.1 acetyl-CoA, 1% (v:v) Triton X-100, and 0.5 oxaloacetate. The absorbance was monitored for 7 min at 25°C and 412 nm.

### Statistical analysis

All data are reported as means±SE. Normality of data was tested with Shapiro-Wilk test. The means were compared using one-way ANOVA and Tukey’s *post hoc* test, when necessary. Regression analyses were used to predict mRNA levels from cardiac tissue lactate concentration expression. Differences were considered significant when P<0.05.

## Results

### Functional capacity and submaximal running performance

Smax and submaximal treadmill running speed reached by the animals of the groups (control, 30, 60, 90%, 0.5 mM CINN, 90% 0.5 mM CINN, 5 mM CINN, and 90% 5 mM CINN) are shown in Figure 1. There were no differences in Smax reached in the graded treadmill exercise test among groups (Figure 1B), showing that the exercise capacity was similar among groups at the beginning of the protocol. As expected, the speeds of the submaximal acute running bouts were significantly different between groups. The treatment with CINN did not change submaximal running performance (Figure 1C).

### Lactate concentration in blood and heart

**Figure 2:**
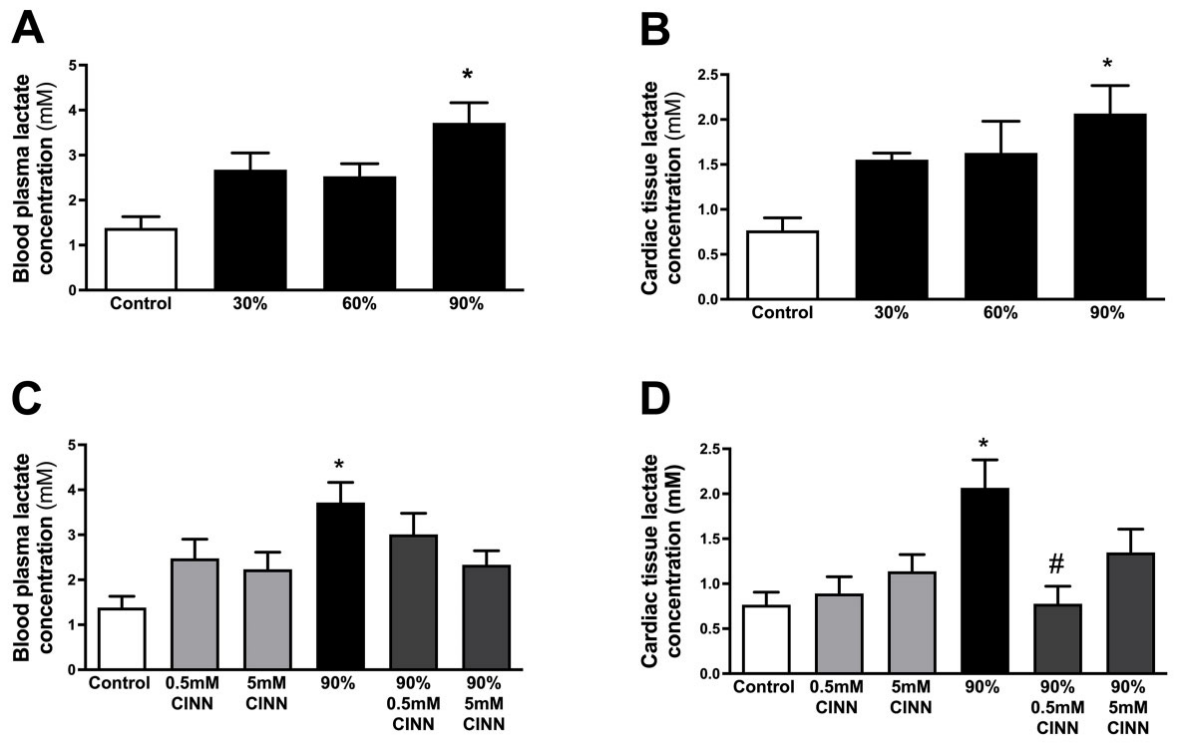
Blood and cardiac lactate concentrations in physical exercise. Blood plasma (**A**) and cardiac tissue (**B**) lactate concentrations of the control and groups running at 30%, 60%, and 90% of maximal speed. Blood plasma (**C**) and cardiac tissue (**D**) lactate levels of the 90%, 0.5 mM CINN, 90% 0.5 mM CINN, 5 mM CINN, and 90% 5 mM CINN groups. Data are reported as means±SE. *P<0.05 *vs* control, ^#^P<0.05 *vs* 90% group (ANOVA). CINN: α-cyano-4-hydroxycynnamate.

In order to verify the effect of different intensities of acute running sessions with constant load (30, 60, and 90% of Smax) on the lactate kinetics, both blood plasma and cardiac tissue were measured at rest and at the end of the submaximal running sessions. There was no significant difference in resting blood lactate levels among groups (control: 1.3±0.2 mM; 30% group: 1.2±0.2 mM; 60% group: 0.9 ±0.1 mM; 90% group: 1.0±0.1 mM; 0.5 mM CINN group: 1.2±0.1 mM; 90% 0.5 mM CINN group: 1.6±0.1 mM; 5 mM CINN group: 1.3±0.2 mM; 90% 5 mM CINN group: 1.0±0.1 mM). The lactate concentration increased significantly in both blood plasma and cardiac tissue in mice running only at 90% of Smax (Figure 2A and B). Treatment with CINN (0.5 or 5 mM) did not significantly alter blood lactate levels in the group that did not perform exercise and in the group that ran at 90% of Smax (Figure 2C). In contrast, 0.5 mM of CINN significantly inhibited the increase of cardiac lactate concentration only in the group that ran at 90% of Smax (Figure 2D).

We observed that: *i*) increased cardiac lactate concentration was restricted to mice that ran at 90% of Smax; *ii*) 0.5 mM of CINN was the dose that decreased the cardiac lactate concentration; and *iii*) 0.5 mM of CINN did not alter cardiac lactate levels in the group that did not perform any physical exercise. Therefore, we decided to carry out the enzymatic activity and gene expression assays only with the control, 90%, and 90% 0.5 mM CINN groups.

### Effect of exercise intensity and lactate accumulation on the glycolytic and oxidative enzyme activity in cardiac tissue

**Figure 3:**
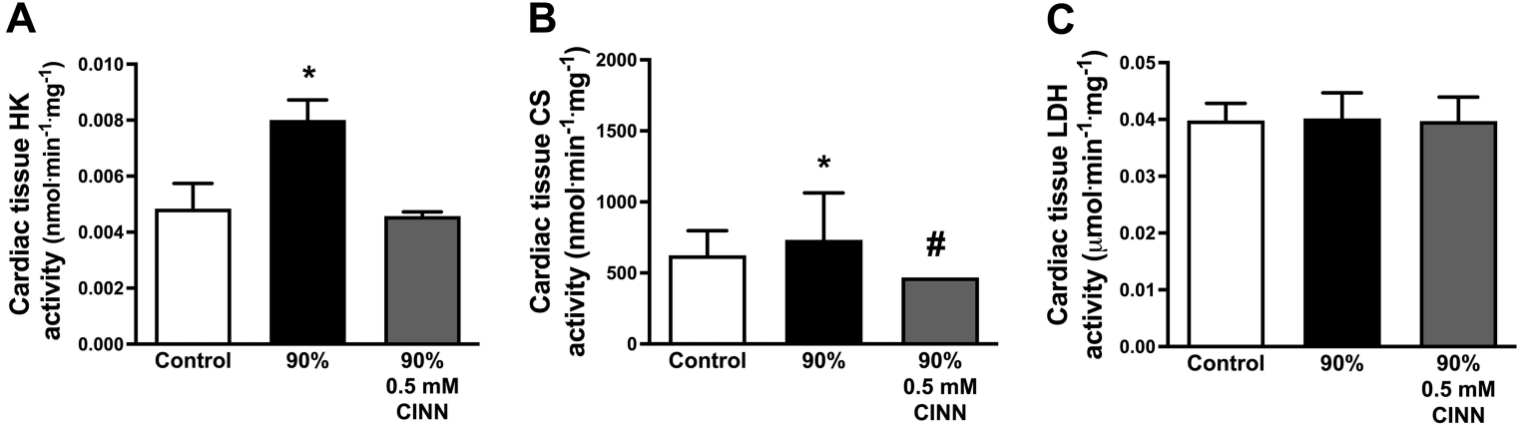
Glycolytic and oxidative enzyme activities in cardiac tissue. The activity of hexokinase (HK) (**A**), citrate synthase (CS) (**B**), and lactate dehydrogenase (LDH) (**C**) in animals of the control group and groups running at 90% of maximal speed and at 90% plus 0.5 mM CINN (α-cyano-4-hydroxycynnamate). Data are reported as means±SE. *P<0.05 *vs* control, ^#^P<0.05 *vs* 90% (ANOVA).

The activity of the enzymes HK and CS was increased in the 90% group compared with the control group (Figure 3A and B). CS activity was significantly reduced in the group that ran at 90% of Smax and received 0.5 mM of CINN (Figure 3B). However, no significant changes were observed in LDH activity (Figure 3C).

### Lactate modulated the expression of oxidative metabolism genes in cardiac tissue

**Figure 4:**
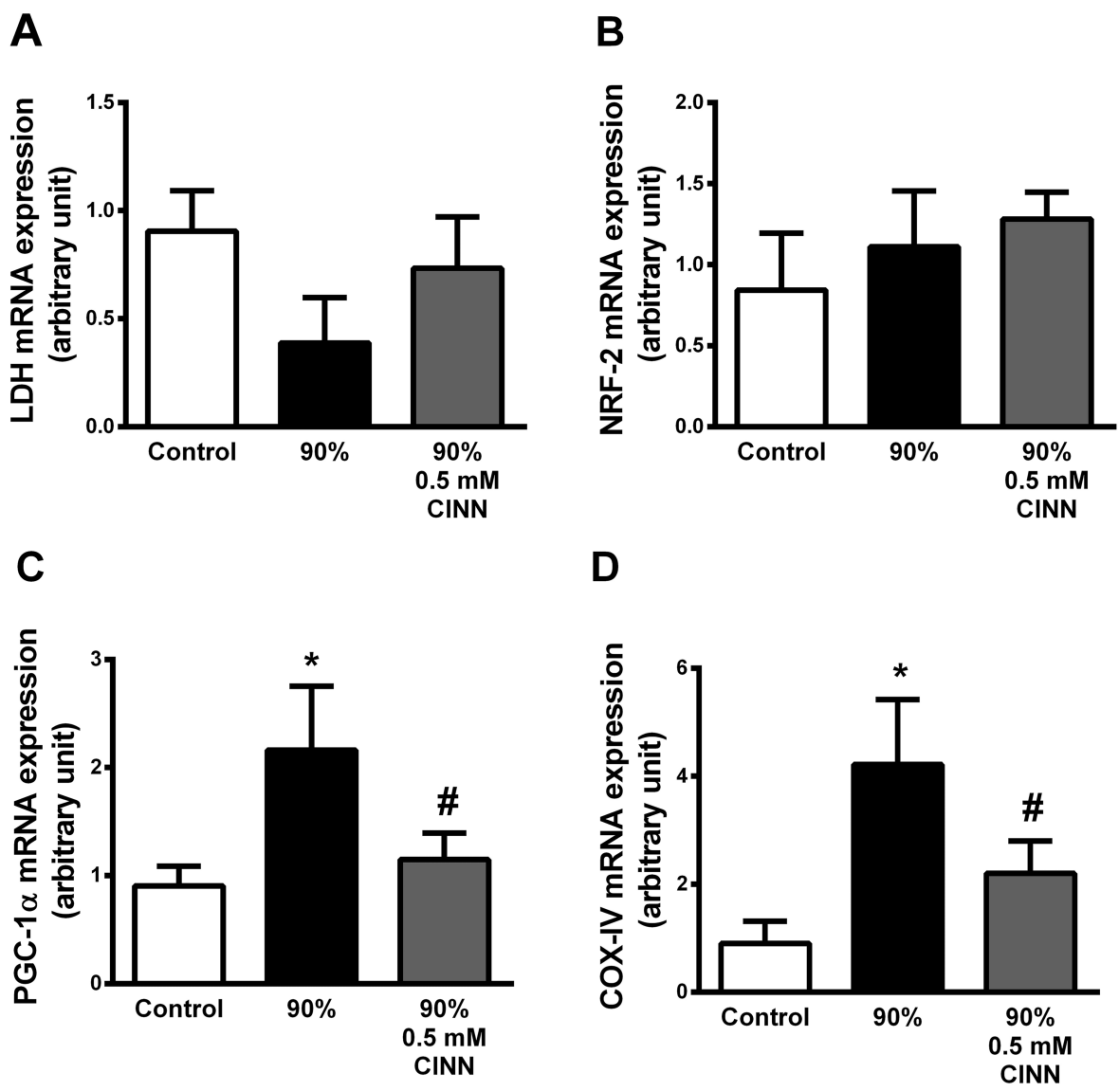
Expression of genes related to oxidative and glycolytic metabolism in cardiac tissue. Lactate dehydrogenase (*LDH*) (**A**), nuclear factor erythroid 2-related factor 2- (*NRF-2*) (**B**), peroxisome proliferator-activated receptor gamma coactivator 1-alpha (*PGC-1α*) (**C**), and cytochrome oxidase IV (*COX-IV*) (**D**) mRNA levels in the control group and groups running at 90% maximal speed and 90% plus 0.5 mM CINN (α-cyano-4-hydroxycynnamate). Data are reported as means±SE. *P<0.05 *vs* control, ^#^P<0.05 *vs* 90% groups (ANOVA).

We observed that there was no significant change in the *LDH* and *NRF-2* mRNA levels in groups, regardless of the 0.5 mM CINN treatment (Figure 4A and B). In contrast, both *COX-IV* and *PGC-1α* mRNA levels were increased in mice running at 90% of Smax. This response was reduced in the group under CINN treatment (Figure 4C and D).

**Figure 5:**
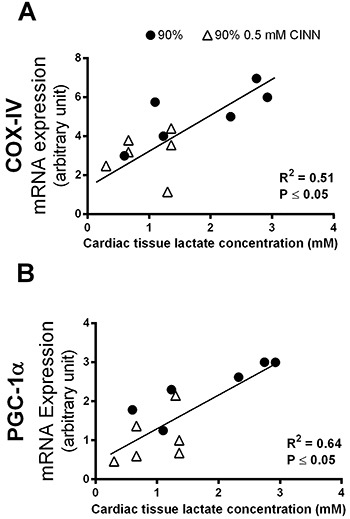
Cytochrome oxidase IV (*COX-IV*) (**A**) and (**B**) peroxisome proliferator-activated receptor gamma coactivator 1-alpha (*PGC1-α*) gene expression correlation with cardiac lactate concentration in animals running at 90% maximal speed and at 90% plus 0.5 mM CINN (α-cyano-4-hydroxycynnamate).

To investigate the relationship between lactate and gene expression in cardiac tissue, a regression analysis was performed for *COX-IV* and *PGC-1α* mRNA levels (Figure 5A and B). Both *COX-IV* and *PGC-1α* mRNA levels were positively and significantly correlated with the cardiac lactate concentrations.

## Discussion

The present study demonstrated that an acute bout of exercise at high intensity triggered an increase in cardiac tissue lactate levels, which was associated with up-regulated *PGC1-α* and *COX-IV* mRNA levels.

It is well established that both blood and cardiac lactate levels increase during physical exercise, especially at high intensities (7,17). In this study, we submitted the animals to a high-intensity acute exercise on a treadmill with or without previous administration of CINN, a MCT blocker, to alter the cardiac lactate concentration and verify the associated modulation of gene expression. Since there were no previous data that tested CINN administration *in vivo* and we were unable to measure the pharmacokinetic properties of the drug (absorption and distribution) and drug/receptor interactions when administered via *ip*, we decided to set the concentration of CINN that would be able to prevent the increase of cardiac tissue lactate levels induced by exercise (90% of Smax). Therefore, two concentrations of the drug (0.5 and 5 mM) were tested based on the previous values of IC_50_ and efficacy obtained for inhibition of lactate transport *in vitro* (14-16). We have also tested the effect of CINN on blood and cardiac lactate levels in animals that did not perform exercise.

The data showed that all groups reached similar Smax in the graded treadmill exercise test, confirming that they displayed comparable exercise performance. As expected, exercise performance was not influenced by CINN administration. Although one might argue that CINN would impair gluconeogenesis and reduce exercise performance of the 90% group, we believe that although gluconeogenesis might be compromised in the animals submitted to high-intensity acute running, it would not be able to reduce exercise performance. This is because most of the lactate synthesized might be oxidized as an energy substrate (2). Furthermore, the animals were only able to run for about 10 min, a duration that any decrease in skeletal muscle glycogen stocks might be limited and buffered by liver glycogenolysis (18,19). Finally, the animals did not perform the exercise in a fasting state since they had free access to standard chow, implying that skeletal muscle glucogenic stocks were not depleted before exercise. Animals that ran at 30 and 60% of Smax were able to exercise much longer (∼60 min) than those that ran at 90% of Smax, which reached fatigue at around 10 min of exercise (data not shown). Although it is tempting to assume a cause-and-effect relationship between increased lactate levels and reduced exercise performance, the accumulated data in the literature support that these are parallel events (3,20,21). Therefore, it seems more reasonable to suggest that the reduced exercise performance at the highest running intensity (90% of Smax) was related to other mechanisms (i.e., the central nervous system fatigue or synthesis of inorganic phosphate in skeletal fibers) rather than to the accumulation of lactate in skeletal muscles and blood (2,21-24).

Only the 90% group showed a significant increase in blood lactate levels compared to the control group. This result was in accordance with previous data from Ferreira et al. (13), who showed that maximal lactate steady state in mice corresponds to an intensity of 60% Smax. At this speed, lactate blood concentration is maintained overtime without continued accumulation. The increase in blood lactate levels at 90% of Smax occurred parallel to the increase in cardiac lactate levels. This phenomenon can be explained by the extracellular lactate shuttle hypothesis, which states that lactate produced in skeletal muscle fibers (especially in type II fibers) is transported to other organs, such as the heart, to be used as energy substrate (1,2).

Although we expected that CINN might block the distribution of lactate to other tissues/cells (e.g., red blood cells, liver, brain, etc.) and block its transport from skeletal muscle to bloodstream altering its levels, both doses of CINN did not alter the blood lactate concentration during exercise. A possible explanation for the phenomenon could be related to the differences observed in the expression of MCT isoforms among tissues and their affinity for CINN. In fact, MCT1 isoform is expressed ubiquitously in different tissues, but mainly in heart tissue, erythrocytes, type I skeletal muscle fibers, and neurons (6,25). On the other hand, MCT4 is expressed in type II skeletal muscle fibers, white blood cells, and astrocytes (4,5,8). According to the experiments conducted by Fox et al. (26), the affinity of CINN for MCT1 is higher than for MCT4. This would explain why the lactate release from skeletal muscle fibers was not impaired during high intensity exercise. CINN (0.5 and 5 mM) administration had no effect on blood lactate levels of mice that remained at rest.

On the other hand, the administration of 0.5 mM CINN, but not 5 mM, significantly reduced cardiac lactate concentration during physical exercise. CINN had no effect on lactate concentration of cardiac tissue of animals that did not perform physical exercise. In other words, only the dose of 0.5 mM CINN was effective during exercise. According to previous data, lactate transport in isolated cardiac cells is mostly mediated by MCT1 (5). Since MCT1 has higher affinity for CINN than MCT4 (isoform expressed in type II skeletal muscle fiber membranes) (26), cardiac lactate levels would be selectively affected by CINN administration. On the other hand, the highest dose of the drug had no effect on cardiac lactate. Although at first glance this might be contradictory, we cannot forget that LDH is the enzyme responsible for the turnover of pyruvate into lactate. It works near equilibrium, which means that the substrate concentration regulates the direction of the reaction (2). The cardiac lactate isoforms mostly oxidize lactate into pyruvate, especially during physical exercise when the influx of lactate into the cells is highest (27). However, Chatham et al. (28) showed that cardiac cells also synthesize lactate, even during physical exercise. Cardiac lactate transport seems to be highly inhibited by 5 mM CINN, approximately 70% of this transport when lactate concentration was 2 mM (15) and 90% when lactate concentration was 0.5 mM (16). Therefore, the administration of 5 mM CINN in the present study might have led to inhibition of lactate influx into cardiac cells and driven the synthesis of lactate, preventing the decrease of its levels in cytoplasm.

The data obtained from RT-PCR experiments showed that mRNA levels of *PGC-1α* and *COX-IV* were up-regulated in the 90% group compared to the 90% 0.5 mM CINN group, but the expression of other genes did not differ among the groups. In order to evaluate the relationship between cardiac lactate levels and expression of *PGC1-α* and *COX-IV* genes, a regression analysis was obtained using data from the 90% group and the 90% 0.5 mM CINN group. In both cases, lactate concentration was significantly and positively correlated with mRNA levels, suggesting that lactate may modulate *PGC1-α* and *COX-IV* at transcriptional levels *in vivo*. Indeed, some studies have shown that lactate acts as a signaling molecule in different tissues and cells. Lactate *i*) modulates the expression of oxidative metabolism genes and proteins in L6 cell lineage (11) and in the plantaris and the soleus muscles (29); *ii*) induces triglycerides storage and increases the expression of MCT1/4 and GPR81 receptors as well as the activities of the enzymes malonyl CoA:ACP transferase and pyruvate dehydrogenase in C2C12 myotubes (30); *iii*) increases skeletal muscle mass and stimulates muscle regeneration, both in cells and *in vivo* by its exogenous administration (31-33); and *iv*) stimulates the expression of the LOC genes in perfused hearts (12). One potential mechanism that links lactate concentration to *COX-IV* and *PGC1-α* mRNA levels is its capacity to increase synthesis of reactive oxygen species (ROS) in cardiac tissue by activating NADH oxidase activity (12). Increased ROS levels modulate AMPK activity and Ca^2+^ handling. AMPK induces NFκB and NRF-1/2 translocation to the nucleus and enhances *PGC1-α* and *COX-IV* expression (34). On the other hand, Ca^2+^-dependent activated kinases increase AMPK phosphorylation and also modulate *PGC1-α* expression (35). Further experiments should be conducted to verify this hypothesis.

Our study is the first to demonstrate the association of the endogenous production of lactate during an acute bout of physical exercise and cardiac gene expression *in vivo*. In fact, it is well recognized that exercise acutely and chronically up-regulates oxidative metabolism components in skeletal muscle (36,37) and in the heart (38). We suggest that lactate may be, in part, a mediator of those exercise-induced cardiac adaptations. Recent data support the hypothesis that metabolic intermediaries may also act as signaling molecules, altering protein levels, and influencing cell phenotypes (39). The present study demonstrated the effect of altered lactate levels immediately after an acute exercise bout. Further experiments should be carried out to investigate if lactate directly modulates cardiac gene expression or if they are only associated events that occur in parallel. It would be interesting to determine the effect of lactate over prolonged periods or even the response observed in accumulated physical exercise sessions (chronic exercise).

As expected, HK and CS maximal activities were increased in the 90% group, probably because of the increase in energy requirement during intense exercise. The increase of the sympathetic drive and substrate flow (glucose, lactate, and free fatty acid) helps sustain ATP levels and maintain cardiac output during physical exercise as it increases glycolysis and glycogenolysis in various cells. (40). Although, free fatty acids are not the main substrate of cardiac cells during high-intensity exercise, both the increase of sympathetic flow and decrease of insulin stimulate adipose and cardiac tissues lipolysis elevating fatty acid concentrations and its oxidation in cardiac cells (40). On the other hand, CINN (0.5 mM) reduced the activity of CS and HK, suggesting that blocking lactate uptake may impact the activity of these enzymes. However, LDH was not altered by the MCT blocker, suggesting that CINN could act blocking the MCT1 channels expressed in the inner membrane of mitochondria (1,2). Nevertheless, this should be investigated in future studies.

### Conclusion

Our study showed that lactate was associated to transcription of cardiac genes involved in oxidative metabolism and mitochondrial biogenesis. Lactate is an important substrate for cardiac cells, and influences energy supply *in vivo*. Oxidation of lactate by cardiomyocytes is more efficient and is up-regulated during exercise. Data have shown that it also acts as a signaling molecule *in vitro*, influencing protein and enzymatic profiles of different cell types (such as cardiac). However, to our knowledge, this is the first work that demonstrated that lactate may act as a signaling molecule during acute high-intensity exercise, providing evidence of its important role in exercise-induced adaptations of cardiac metabolism. Further investigations might clarify which of these adaptations are related to lactate increase and their implications to cardiac performance during physical exercise.
